# Current status of malnutrition, frailty, and sarcopenia risks in cancer patients with comorbidities

**DOI:** 10.3389/fpubh.2026.1820491

**Published:** 2026-06-24

**Authors:** Lixue Cui, Yu Zhang, Guoshan Gao, Xiaoyan Hao, Ronghuan Zhang, Ling Zhou, Xirui Jiang, Lili Zhao

**Affiliations:** 1 School of Nursing, Ningxia Medical University, Yinchuan, Ningxia, China; 2 Department of Emergency, People's Hospital of Ningxia Hui Autonomous Region, Ningxia Medical University, Yinchuan, Ningxia, China; 3 Department of Cardiac and Vascular Surgery, People's Hospital of Ningxia Hui Autonomous Region, Ningxia Medical University, Yinchuan, Ningxia, China; 4 Department of Nursing, People's Hospital of Ningxia Hui Autonomous Region, Ningxia Medical University, Yinchuan, Ningxia, China

**Keywords:** frailty, malnutrition, modified Poisson regression, patients with comorbid cancer, sarcopenia

## Abstract

**Objective:**

To analyze the coexistence of malnutrition, frailty, and sarcopenia in patients with cancer comorbid.

**Methods:**

Using convenience sampling, 476 patients with cancer comorbidities were recruited from two Grade III Class A hospitals in the Ningxia Hui Autonomous Region between January 2025 and December 2025. Data were collected using a general information questionnaire, the Nutritional Risk Screening 2002 (NRS-2002), the FRAIL frailty scale, and the SARC-Calf sarcopenia screening scale. Univariate analysis and multivariate modified Poisson regression analysis were performed to identify influencing factors, and Venn diagrams were used to illustrate the overlap among the three conditions.

**Results:**

The prevalence rates of malnutrition and sarcopenia were 31.3 and 65.7%, respectively. According to the FRAIL scale, 115 patients (24.2%) had no frailty, 245 (51.5%) were classified as pre-frailty, and 116 (24.4%) met the criteria for established frailty. A total of 122 patients (25.6%) presented with all three conditions simultaneously. The Venn diagram showed that frailty and sarcopenia had the highest coexistence rate (55.2%). Multivariable modified Poisson regression analysis revealed that older age (31–60 years and >60 years vs. 18–30 years), advanced tumor stage (Stage II–III and Stage IV vs. Stage I), and rural residence (rural vs. urban) were harmful predictors (PR > 1, *p* < 0.05) of the coexistence of the three conditions, whereas higher socioeconomic support (PR < 1, p < 0.05) was a protective predictor.

**Conclusion:**

The coexistence of malnutrition, frailty, and sarcopenia is common among patients with cancer comorbidities and is significantly associated with age, tumor stage, and socioeconomic factors. Implications for nursing practice: Primary care facilities should routinely monitor nutritional status, frailty, and sarcopenia in older adults (aged ≥ 65 years) with cancer and comorbidities, while also establishing a tiered intervention system and a referral mechanism to higher-level hospitals when necessary.

## 1 Introduction

Cancer comorbidity is prevalent among older adults patients, with over 50% having at least one chronic condition and approximately one-quarter having four or more (1, 2). Large-scale cohort studies have confirmed significant bidirectional associations between cancer and chronic diseases such as cardiovascular disease and dementia, and the prevalence of multiple coexisting conditions continues to rise (3). The problem of malnutrition in patients with cancer comorbidities has expanded from a single dimension to an overlapping syndrome that includes frailty and sarcopenia. These three conditions share similar phenotypes (e.g., weight loss and functional decline) but differ in etiology and have complex synergistic relationships—malnutrition can accelerate the development of frailty and sarcopenia, and vice versa (4). The ESPEN guidelines have collectively classified them as nutrition-related conditions (5). The diagnostic criteria for frailty, sarcopenia, and malnutrition overlap with respect to weight loss, reduced muscle mass, and decreased muscle strength (6). An increase in body fat accompanied by a decrease in muscle mass can lead to a pro-inflammatory state and immune dysfunction (7). The pro-inflammatory state and immune dysfunction associated with sarcopenia are partially mediated by cytokines such as IL-1, IL-6, and TNF-α, as well as the activation of NF-κB and MAPK signaling pathways (8). These same inflammatory mechanisms may also underlie the bidirectional association between sarcopenia and cancer. Clinical outcomes of cancer treatment may be compromised by the development of malnutrition and metabolic disorders, not only due to the physical and metabolic effects of cancer itself, but also as a result of anticancer therapies (9). Recent evidence suggests that insulin receptor signaling may promote cancer progression by modulating inflammatory pathways, further supporting the link between metabolic disorders and pro-tumor inflammation in patients with cancer comorbidities (10). Previous meta-analyses have found that 49.7% of individuals concurrently present with both frailty and malnutrition, while 41.6% exhibit both sarcopenia and malnutrition (11). However, domestic studies have predominantly focused on single or pairwise associations (12, 13), lacking a systematic exploration of the overlap among all three conditions. Although research in other countries has gradually begun to treat this as a syndrome requiring integrated management (14, 15), there remain few reports on relevant epidemiological data and clinical management strategies in China. Therefore, this study aims to explore the coexistence of malnutrition, frailty, and sarcopenia in hospitalized cancer patients with comorbidities from the perspective of epidemiology and clinical management of overlapping syndromes. By constructing an optimal regression model to analyze influencing factors and using Venn diagrams to visualize overlapping patterns, this study seeks to provide a basis for the early clinical identification of high-risk populations and the formulation of collaborative intervention strategies, thereby reducing resource waste and improving patient prognosis.

## 2 Data and methods

### 2.1 Study subjects

Convenience sampling was used to recruit 476 patients with cancer comorbidities from the oncology, gastrointestinal surgery, urology, and respiratory and critical care medicine departments of two Grade III Class A hospitals in the Ningxia Hui Autonomous Region between January 2025 and December 2025. Inclusion criteria: (1) Pathologically confirmed diagnosis of cancer with an estimated life expectancy of ≥6 months; (2) Age ≥18 years; (3) Presence of one or more chronic diseases in addition to cancer. Chronic diseases were identified based on the International Classification of Diseases (ICD-10) standards, recording the names of diagnosed chronic diseases from electronic medical records and patient/family interviews. Patients with a BMI < 18.5 kg/m^2^ and an NRS-2002 nutritional risk screening score≥3 were diagnosed with malnutrition and underwent severity grading; (4) Adequate cognitive ability and cooperation; (5) Voluntary participation with informed consent.

### 2.2 Methods

#### 2.2.1 General clinical data

A clinical database was established to record the following information for each study subject: (1) General demographic data: age, sex, education level, marital status, occupation, income level, place of residence, ethnicity, and whether living alone; (2) Disease status: cancer/tumor type, stage, and duration of illness; (3) Treatment plan: including surgery, radiotherapy, chemotherapy, targeted therapy, etc.; (4) Anthropometric indicators: height, weight, upper arm circumference, calf circumference, dominant hand grip strength, as well as inquiry regarding the patient’s weight changes over the past 6 months and 1 year; (5) Laboratory indicators: hemoglobin (Hb), albumin (Alb), white blood cell count (WBC), and C-reactive protein (CRP).

#### 2.2.2 Malnutrition assessment

Malnutrition Grading: The NRS-2002 Nutritional Screening Form consists of three parts: (1) Disease score; (2) Nutritional status score; ③ Age score. If the NRS-2002 score is ≥3, it indicates that the patient is at nutritional risk. For patients identified as being at risk of malnutrition based on electronic medical records, malnutrition is diagnosed according to the Global Leadership Initiative on Malnutrition (GLIM) criteria and classified by severity. GLIM includes phenotypic and etiological diagnostic criteria. Phenotypic indicators include: involuntary weight loss, low BMI, and reduced muscle mass; etiological indicators include: reduced food intake or absorption, and disease burden/inflammation. Among screening-positive patients, at least one criterion from each of the two categories must be met to assess malnutrition.

#### 2.2.3 Assessment of frailty

The FRAIL scale, developed by American scholar Morley (16), consists of five items. Each item is scored as 1 point, with total scores ranging from 0 to 5. A score of 0 indicates no frailty, 1–2 indicates pre-frailty, and 3–5 indicates frailty. This scale has been used in China with good reliability and validity, with a Cronbach’s *α* coefficient of 0.705 (17).

#### 2.2.4 Assessment of sarcopenia

The SARC-Calf score was used, which was modified by Brazilian scholar Barbosa-Silva et al. (18). The components include muscle strength, walking assistance, seat-to-stand, stair climbing, and number of falls, each worth 2 points, with the addition of an objective measurement indicator—calf circumference (CC). For women, a CC ≤ 33 cm receives 10 points, while a CC > 33 cm receives 0 points. For men, the cutoff is 34 cm, with the same scoring method. Chinese scholar Huang et al. (19) translated the questionnaire into Chinese, and it has since been widely used. The total score ranges from 0 to 20, and a score ≥ 11 indicates a higher risk of sarcopenia.

### 2.3 Data collection and quality control methods

This study investigated the current status of malnutrition, frailty, sarcopenia risk, and cachexia in patients with cancer comorbidities, and analyzed the overlap among these four conditions. Prior to data collection, the researchers received standardized training. During data collection, the purpose of the study and questionnaire completion instructions were fully explained to patients, and questionnaires were distributed after obtaining informed consent. General patient information was collected through medical record reviews and in-person interviews. Disease history and biochemical indicators were obtained from medical records. During questionnaire completion, standardized instructions were used to address patient inquiries. Questionnaires were distributed, completed, checked, and supplemented as necessary on site. A total of 488 questionnaires were distributed, and 476 valid questionnaires were collected, yielding a valid response rate of 97.5%.

### 2.4 Statistical methods

Statistical analyses were performed using R version 4.3.1 with RStudio as the integrated development environment. Given the cross-sectional design and the high prevalence of all three outcomes (malnutrition: 31.3%, frailty: 75.8%, sarcopenia: 65.7%), the odds ratio (OR) derived from logistic regression would substantially overestimate the true effect size, as demonstrated by Barros and Hirakata (20). Therefore, we directly estimated prevalence ratios (PR) using a modified Poisson regression approach with a robust error variance (sandwich estimator), as proposed by Zou (21) and recommended by Barros & Hirakata. This approach fits a generalized linear model (GLM) with a Poisson distribution and a log link function; the use of robust standard errors corrects for the misspecification of the variance when applying Poisson regression to binary data. All categorical variables were entered into the modified Poisson regression models as dummy-coded variables, each with a predefined reference category. Reference categories were selected based on clinical and epidemiological rationale, representing the lowest expected risk or the most favorable clinical status (e.g., early-stage cancer, youngest age group, or female gender). For each categorical variable, prevalence ratios (PRs) for non-reference categories were estimated relative to the reference category. Age was categorized into three groups (18–30, 31–60, and >60 years) to maintain consistency with the univariate analysis. All models were adjusted for potential confounders selected based on univariate analysis (*p* < 0.05), and reference categories for all categorical variables were explicitly indicated in the results table (Table 1). Two-sided tests were performed with a significance level set at *p* < 0.05. The glm function in the stats package with robust standard errors from the sandwich package was used for modified Poisson regression. The VennDiagram package (version 1.7.3) was used to generate Venn diagrams, and the Akaike information criterion (AIC) was calculated for model selection.

**Table 1:** Multivariable modified Poisson regression analysis for Prevalence Ratios (PR) of malnutrition, frailty, and sarcopenia.

Outcome and variable	Category	Reference	PR value	95%CI	*p* value
Risk of malnutrition					
Gender	Male	Female	0.85	0.270–0.945	<0.001
Age	31–60 years	18–30 years	1.35	1.101–1.654	0.005
	>60 years	18–30 years	1.55	1.283–1.889	<0.001
Education level	Junior/senior high school	Primary or below	0.94	0.404–0.984	0.013
	College and above	Primary or below	0.82	0.723–0.934	0.002
Income level	1,001–3,000 yuan	<1,000 yuan	0.82	0.352–0.874	0.015
	3,001–5,000 yuan	<1,000 yuan	0.80	0.663–0.978	0.002
Insurance type	No	Yes	1.29	1.008–2.962	0.050
Cancer type	Breast/thyroid	Other types	0.62	0.405–0.956	0.001
TNM staging	Stage II–III	Stage I	1.28	1.022–2.537	0.045
	Stage IV	Stage I	1.56	1.240–1.960	0.034
Risk of frailty					
Age	31–60 years	18–30 years	1.32	1.087–1.624	0.008
	>60 years	18–30 years	1.50	1.220–1.842	<0.001
Employment status	Unemployed	Gov/Enterprise	1.12	0.957–1.464	0.156
	Retired	Gov/Enterprise	1.34	1.124–1.687	0.048
	Farming/Odd jobs	Gov/Enterprise	1.15	1.020–1.413	0.029
Insurance type	No	Yes	1.18	1.139–1.298	0.008
Risk of sarcopenia					
Gender	Male	Female	0.99	0.397–1.027	0.067
Age	31–60 years	18–30 years	1.30	1.061–1.628	0.012
	>60 years	18–30 years	1.52	1.267–1.839	<0.001
Education level	Junior/senior high school	Primary or below	0.98	0.925–1.047	0.072
	College and above	Primary or below	0.95	0.918–1.025	0.115
Employment status	Farming/odd jobs	Government/enterprise	0.92	0.655–0.977	0.029
Income level	1,001–3,000 yuan	<1,000 yuan	1.22	0.934–1.955	0.115
	3,001–5,000 yuan	<1,000 yuan	1.13	0.865–1.573	0.075
Residential area	Rural	Urban	1.17	1.005–2.214	0.047
Living alone	Yes	No	1.25	1.130–1.525	<0.001

*PR, prevalence ratio; CI, confidence interval. All models were adjusted for potential confounders that were statistically significant in univariate analyses (*p* < 0.05). Reference categories were selected based on clinical criteria to represent the lowest expected risk or most favorable status. Age was categorized consistently with the univariate analysis.

## 3 Results

### 3.1 General information of patients with cancer comorbidities

A total of 488 questionnaires were distributed in this study. Eleven questionnaires with obvious logical errors were excluded, yielding 476 valid questionnaires, for a valid response rate of 97.5%. Among the 476 respondents, the mean age was 62.3 ± 12.5 years, with the majority being middle-aged or older adults (62.2% aged 31–60 years), and 67.0% were female. The patients generally had a low level of education (43.7% had primary school education or below), and their income was predominantly low to moderate (63.0% had a monthly income of 1,000–3,000 yuan). In addition, 73.1% had medical insurance. The most common cancer types were lung cancer (21.2%) and colorectal cancer (13.4%), with 65.5% of patients at stages II–III. Detailed information is presented in Table 2.

**Table 2:** General information on patients with comorbid cancer.

Item	Group	Number of cases	Percentage (%)
Malnutrition		149	31.3
Frailty	Pre-frailty	245	51.5
	Frailty	116	24.4
Sarcopenia		313	65.7
Gender	Male	157	33.0
	Female	319	67.0
Age	18–30	4	0.8
	31–60	296	62.2
	Over 60	176	37.0
Level of education	Elementary school and below	208	43.7
	Junior high school and high school	209	43.9
	College and above	59	12.4
Occupation	Farming or odd jobs	150	31.5
	Government agency, enterprise, or public institution	103	21.6
	Unemployed	137	28.8
	Retired	86	18.1
Average monthly income per capita	Less than 1,000 yuan	82	17.2
	1,001–3,000 yuan	300	63.0
	3,001–5,000 yuan	84	19.7
Medical insurance	Yes	348	73.1
	No	128	26.9
Living situation	City	273	57.3
	Rural	203	42.7
Living alone	Yes	98	20.6
	No	378	79.4
Tumor location	Chest tumor (including lung cancer, breast cancer, esophageal cancer, etc.)	157	33.0
	Digestive system tumors (including colorectal cancer, gastric cancer, liver cancer, gallbladder cancer, pancreatic cancer, etc.)	126	26.5
	Genitourinary system tumors (including: prostate cancer, cervical cancer, ovarian cancer, endometrial cancer, bladder cancer, etc.)	100	21.0
	Head and neck tumors (including thyroid cancer, nasopharyngeal cancer, etc.)	40	8.4
	Blood system tumors (including: multiple myeloma, lymphoma, leukemia, etc.)	30	6.3
	Other tumors (including: glioma, pelvic cancer, vaginal cancer, and some unclassified tumors)	23	4.8
TNM staging	Stage I	6	1.3
	Stage II-III	312	65.5
	Stage IV	156	32.8
Duration of illness	≤1 year	79	16.6
	1 to 5 years	261	54.8
	>5 years	136	28.6
Laboratory test results	White blood cells		4.91 ± 1.71
	Albumin		34.86 ± 4.78
	Hemoglobin		112.81 ± 22.62
BMI value	BMI		23.54 ± 4.25

### 3.2 Coexistence of malnutrition, frailty, and sarcopenia in patients with cancer comorbidity

**Figure 1:**
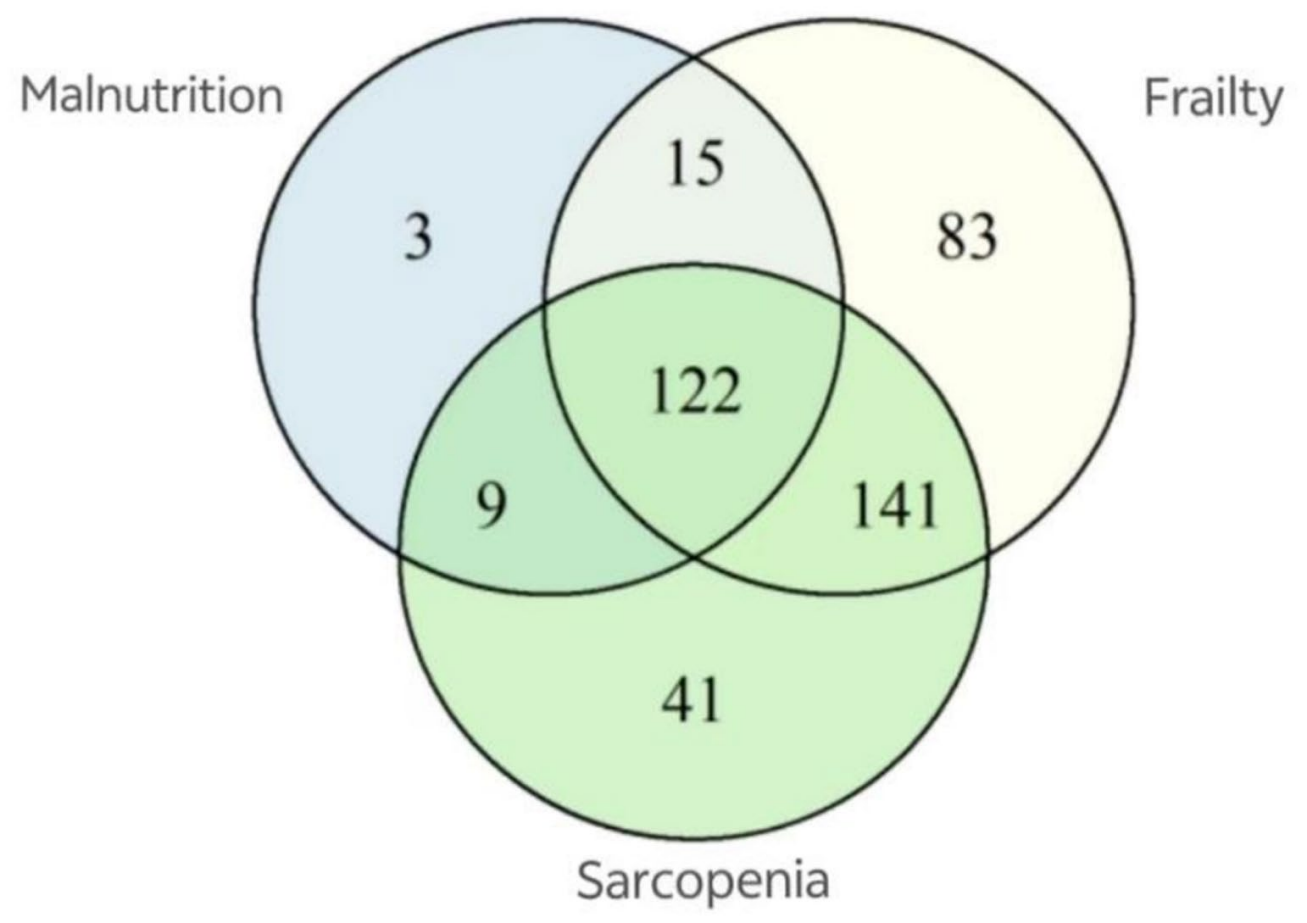
Prevalence of malnutrition risk, frailty, and sarcopenia risk and their overlap (*N* = 476; 62 patients with none of the three conditions are not shown in the overlap region).

The Venn diagram (Figure 1) illustrates the overlap among malnutrition risk, frailty, and sarcopenia risk. Specifically, the prevalence of malnutrition risk was 31.3% (149/476), and the prevalence of sarcopenia risk was 65.7% (313/476). According to the FRAIL scale, 115 patients (24.2%) had no frailty (score 0), 245 patients (51.5%) were classified as pre-frailty (scores 1–2), and 116 patients (24.4%) met the criteria for established frailty (scores 3–5). The prevalence of concurrent risks for all three conditions was 25.6% (122/476). Regarding pairwise coexistence, 137 patients (27.5%) were at risk for both malnutrition and sarcopenia, and 263 patients (55.2%) were at risk for both frailty and sarcopenia (patients with all three risks are included in the pairwise counts).

### 3.3 Univariate analysis of nutritional status, frailty, and sarcopenia risk in patients with cancer comorbidity

The results of the univariate analysis showed that nutritional risk differed statistically by gender, educational level, income level, health insurance status, living arrangements, and living alone (*p* < 0.05); frailty differed statistically by gender, educational level, employment status, income level, health insurance status, and disease duration (*p* < 0.05); sarcopenia showed statistically significant differences in age, occupation, place of residence, and TNM stage (*p* < 0.05), as shown in Table 3.

**Table 3:** Univariate analysis of the risk of malnutrition, frailty, and sarcopenia in patients with cancer comorbidities (*N* = 476).

Variable	Category	Malnutrition risk		Frailty risk		Sarcopenia risk	
		*χ* ^2^	*p*	*χ* ^2^	*p*	*χ* ^2^	*p*
Gender	Male	10.86	<0.001	8.35	<0.001	0.34	0.560
	Female						
Age	18–30 years	47.96	0.000	46.21	0.000	6.92	0.031
	31–60 years						
	>60 years						
Education level	Primary school or below	31.46	<0.001	19.96	<0.001	3.70	0.157
	Junior/Senior high school						
	College degree or above						
Employment status	Government/Enterprise/Institution	2.94	0.402	13.16	0.004	10.37	0.016
	Unemployed						
	Retired						
	Farming or odd jobs						
Monthly income	<1,000 yuan	10.86	0.004	9.43	0.009	5.24	0.073
	1,001–3,000 yuan						
	3,001–5,000 yuan						
Medical insurance	Yes	2.48	0.036	7.36	<0.001	3.33	0.208
	No						
Residence	Urban	5.21	0.034	0.62	0.429	6.08	0.014
	Rural						
Living alone	Yes	2.87	0.035	2.74	0.545	2.43	0.539
	No						
TNM stage	Stage I	0.02	0.988	5.85	0.054	20.09	<0.001
	Stage II–III						
	Stage IV						
Disease duration	≤1 year	2.62	0.270	9.74	0.008	0.14	0.933
	1–5 years						
	>5 years						

*All variables were analyzed using the Chi-square test (*χ*^2^).

### 3.4 Multivariate analysis of malnutrition risk in patients with cancer comorbidities

Multivariable modified Poisson regression analyses were performed to identify factors associated with malnutrition risk, frailty, and sarcopenia. Variables with *p* < 0.05 in univariate analyses were entered into the models, with each outcome as the dependent variable (0 = no, 1 = yes). All categorical variables were dummy-coded with predefined reference categories (see Table 1). For malnutrition risk, harmful predictors (PR > 1, all *p* < 0.05) were older age, lack of medical insurance, and advanced TNM stage; protective predictors (PR < 1, all *p* < 0.05) were female sex, higher education level, higher income (3001–5,000 yuan), and breast/thyroid. For frailty, older age and lack of medical insurance were harmful predictors (PR > 1, all *p* < 0.01). For sarcopenia risk, harmful predictors (PR > 1, all p < 0.05) included older age, farming/odd jobs, rural residence, and living alone. Detailed results are presented in Table 1.

## 4 Discussion

### 4.1 Advanced age, advanced-stage cancer, and social environment are significantly associated with a higher prevalence of the co-occurrence of malnutrition, frailty, and sarcopenia in patients with comorbidities

Multivariable analysis revealed that advancing age and advanced-stage cancer are significantly associated with a higher prevalence of the co-occurrence of these three conditions, consistent with the findings of Meng et al. (22) regarding the cumulative impact of disease severity on frailty. From a mechanistic perspective, older patients often experience metabolic decline and chronic inflammation, while advanced cancer and its treatment can exacerbate muscle protein breakdown and energy expenditure. These pathophysiological processes interact through multiple pathways: the systemic inflammatory response and metabolic disturbances caused by cancer and its treatment synergistically interact with the depletion of physiological reserves resulting from comorbid chronic diseases (23), collectively contributing to a vicious cycle of “malnutrition–sarcopenia–frailty.” Further research has found that socioeconomic factors such as low education level, low income, rural residence, and living alone significantly increase risk, as limited access to resources affects nutritional management, and a lack of social support reduces motivation to maintain healthy behaviors. Based on these findings, older adults patients, those with advanced cancer, and those of low socioeconomic status should be prioritized for screening. Early combined assessment should be conducted using tools such as the FRAIL scale and SARC-Calf score. Primary healthcare resources should be strengthened for rural patients, and community support networks should be established for individuals living alone. A stratified intervention strategy should be developed to provide nutritional subsidies to low-income individuals and to integrate palliative care with nutritional support for patients at the end of life.

### 4.2 Higher socioeconomic support is associated with a lower prevalence of the co-occurrence of malnutrition, frailty, and sarcopenia

Multivariate analysis in this study showed that higher socioeconomic support is associated with a lower prevalence of the co-occurrence of these three conditions, consistent with the findings of previous studies. Patients with higher levels of socioeconomic support may perform better in nutritional management because they pay more attention to obtaining health information and have greater treatment adherence. Higher education levels and income indirectly improve nutritional status and functional maintenance by enhancing health literacy and access to medical resources. Not living alone, as an important indicator of social support, can effectively delay muscle and functional decline by promoting participation in daily activities and emotional interaction. This finding is consistent with the conclusions of Zhang et al. (24) regarding the mechanism by which social support delays frailty. Further research has found that stable employment status and an urban living environment also exhibit protective effects, as better socioeconomic conditions provide improved nutritional support and medical resources, while close social interaction helps maintain physical activity levels. In view of these findings, it is recommended that patients with low levels of social support be included in the key intervention group, strengthening social connections by establishing community support networks and family follow-up systems, and providing group rehabilitation training and psychological intervention for socially isolated patients. The Venn diagram analysis results clearly demonstrate the negative association between social support factors and the prevalence of the three conditions, providing a visual basis for developing targeted intervention strategies. By strengthening the social support system, it is expected that the prevalence of coexisting malnutrition, frailty, and sarcopenia in patients with cancer comorbidities can be significantly reduced.

### 4.3 Impact of clinical heterogeneity on prevalence estimates from tumor type, cancer stage, and treatment modality

The substantial clinical heterogeneity of the study sample with respect to tumor type, cancer stage, and treatment modality may bias the prevalence estimates of malnutrition, frailty, and sarcopenia, thereby limiting the internal validity and generalizability of our findings. Regarding tumor type, patients with thoracic tumors exhibit a significantly higher prevalence of sarcopenia compared to those with hematologic malignancies, likely attributable to a greater systemic inflammatory burden. In contrast, patients with gastrointestinal tumors face a higher risk of malnutrition than those with other tumor types, primarily due to gastrointestinal obstruction or malabsorption. Cancer stage also emerged as a key determinant: the prevalence of malnutrition increases progressively from early-stage to advanced-stage disease. In our sample, patients with Stage IV cancer accounted for 32.8% of the cohort, which may partially explain the relatively high prevalence rates observed (malnutrition: 31.3%; sarcopenia: 65.7%). Treatment modality further influences these outcomes; conventional chemotherapy tends to exacerbate anorexia and fatigue, and has been associated with a significantly higher prevalence of malnutrition compared to immunotherapy (38% vs. 22%, respectively). Owing to the potential for estimation bias arising from the aforementioned sources of heterogeneity, as well as sample size constraints, we were unable to perform stratified analyses by individual tumor type, cancer stage, or treatment modality. Future large-scale, multicenter prospective studies with prespecified stratification or subgroup analyses are warranted to elucidate how clinical heterogeneity affects the prevalence and co-occurrence of these three conditions, ultimately facilitating the development of tumor-specific or stage-specific clinical management pathways.

### 4.4 Co-occurrence of malnutrition, frailty, and sarcopenia is common among patients with comorbid cancer

This study, based on a cross-sectional survey of 476 patients with cancer comorbidities, revealed a high prevalence of the co-occurrence of malnutrition, frailty, and sarcopenia in this population. The results showed that the prevalence rates of these three conditions individually were 31.3, 75.8, and 65.7%, respectively, while the prevalence of their co-occurrence reached 25.6%. A study by Kiss et al. (25) on malnutrition among cancer patients with comorbidities in the United Kingdom found that 11.1% of patients were malnourished, 51.2% exhibited symptoms of frailty, and 6.9% had sarcopenia. The prevalence rates observed in our study are considerably higher than those reported by Kiss et al., which is likely attributable to differences in patient condition severity and age. Our study specifically included older adults patients with advanced cancer during the acute inpatient phase—a population characterized by a hypermetabolic state and low physiological reserve capacity. In contrast, the study by Kiss et al. primarily included younger, community-dwelling patients with early-stage cancer who were in relatively good functional condition. These fundamental differences in baseline characteristics likely explain the observed discrepancies in prevalence estimates. Furthermore, our results indicate that sex, age, educational level, income, health insurance coverage, living arrangements, and living alone were significantly associated with malnutrition in the multivariable analysis. These differences may stem from factors such as lifestyle habits, declining physiological function, insufficient health literacy, and limited access to medical resources. Sex, educational level, employment status, income level, health insurance coverage, and disease duration are all independently associated with frailty. Frailty may be more likely to occur in the context of insufficient social support. Age, living arrangements, and TNM stage were independently associated with sarcopenia, which may be attributed to age-related muscle loss, reduced physical activity among patients, and worsening cachexia in advanced-stage cancer.

Venn diagram analysis visually illustrates significant overlap among these conditions, with the highest coexistence rate (55.2%) observed between frailty and sarcopenia, suggesting a close association among the three. Multivariate analysis further identified advanced age, advanced tumor stage, low educational level, low income, rural residence, and living alone as harmful predictors, whereas female sex, higher socioeconomic status, and social support were associated with protective effects. This indicates that, in addition to disease-specific factors, socioeconomic dimensions play a critical role in underlying disease risk. In clinical practice, early combined screening mechanisms should be established for older adults patients, those with advanced-stage cancer, and those with limited resources, and comprehensive management strategies integrating nutritional support, resistance exercise, and psychosocial interventions should be implemented to break the vicious cycle of “malnutrition–sarcopenia–frailty.”

## 5 Conclusion

The coexistence of malnutrition, frailty, and sarcopenia is particularly prominent among patients with cancer and comorbidities, and is primarily influenced by advanced age, advanced cancer stage, and insufficient social support. It is recommended that primary care facilities routinely monitor nutritional status, frailty, and sarcopenia in older adults patients with cancer and comorbidities, establish a tiered intervention system, and develop referral mechanisms to higher-level hospitals when necessary. Given the constraints of the study timeline and the practical challenges of measurement compliance, this study currently employs a cross-sectional design. Furthermore, as the sample was drawn from two hospitals in Ningxia, selection bias may be present. Future prospective cohort studies should be conducted to further explore the dynamic evolution of these three conditions and the effectiveness of interventions.

## Data Availability

The raw data supporting the conclusions of this article will be made available by the authors, without undue reservation.
